# Characterization of patients with IgA nephropathy with and without associated minimal change disease

**DOI:** 10.3389/fneph.2023.1105933

**Published:** 2023-02-16

**Authors:** Wei-yi Guo, Li-jun Sun, Hong-rui Dong, Guo-qin Wang, Xiao-yi Xu, Wen-rong Cheng, Zhi-rui Zhao, Nan Ye, Yun Liu, Hong Cheng

**Affiliations:** ^1^ Renal Division, Department of Medicine, Beijing Anzhen Hospital, Capital Medical University, Beijing, China; ^2^ Division of Nephrology, Affiliated Hospital of Chifeng University, Chifeng, Inner Mongolia, China

**Keywords:** IgA nephropathy, minimal changed disease, Gd-IgA1, complement C3, characteristics

## Abstract

**Introduction:**

Immunoglobulin A nephropathy (IgAN) presents various clinical manifestations and pathological phenotypes. Approximately 5% of patients with IgAN present with early onset nephrotic syndrome, mild mesangial lesions, and diffuse foot process effacement of podocytes, which resemble minimal change disease (MCD). These patients are defined as MCD-IgAN. Whether MCD-IgAN is a special type of IgAN or simply MCD accompanied by IgA deposition remains controversial.

**Methods:**

A total of 51 patients diagnosed with MCD-IgAN at Beijing Anzhen Hospital from January 2010 to September 2022 were recruited. The clinical and pathological characteristics of IgA-MCD were analyzed. Patients with IgAN but without MCD (non-MCD-IgAN) and healthy participants were enrolled as controls. Galactose-deficient immunoglobulin A1 (Gd-IgA1) and complement C3 were detected both in the circulation and in renal tissues.

**Results:**

We found that the levels of serum Gd-IgA1 were lower in participants with MCD-IgAN than in those with non-MCD-IgAN, but higher than in healthy participants. Gd-IgA1 was rarely deposited in the glomeruli of participants with MCD-IgAN, with a positive rate of only 13.7% (7/51); in contrast, the positive rate in participants with non-MCD-IgAN was 82.4% (42/51). Among renal Gd-IgA1-positive patients, Gd-IgA1 and immunoglobulin A (IgA) colocalized along the glomerular mesangial and capillary areas. Interestingly, we found that the circulating levels of complement C3 were significantly higher in participants with MCD-IgAN than in participants with non-MCD-IgAN. In addition, the intensity of C3c in glomeruli in participants with MCD-IgAN was significantly weaker than in participants with non-MCD-IgAN.

**Conclusions:**

Our study suggests that, in MCD-IgAN, most of the IgA that is deposited on glomeruli is not the same pathogenic Gd-IgA1 as found in general IgAN. Complement activation both in the circulation and in the renal locality was much weaker in MCD-IgAN than in non-MCD-IgAN. Our study suggests that IgAN with MCD might be MCD with coincidental IgA deposition.

## Introduction

Immunoglobulin A nephropathy (IgAN) is the most frequent form of primary glomerulonephritis worldwide (1). Up to 20%–40% of patients will have reached end-stage kidney disease approximately 20 years after diagnosis (2). The clinical presentation and histologic features of IgAN are highly variable (3). In addition to the classic presentation (3), such as recurrent episodes of gross hematuria after mucosal infections, some patients with IgAN present with onset nephrotic syndrome, mild mesangial lesions under light microscopy, and diffuse foot process effacement of podocytes under electron microscopy, which resemble minimal change disease (MCD) (4, 5). These patients are defined as MCD-IgAN. Some studies have reported these patients to have a special clinical subtype of IgAN (5, 6), whereas others have considered this to be MCD accompanied by IgA deposition (7, 8).

Today, the pathophysiology of IgAN is considered to be a multi-hit mechanism (9). The elevated serum levels of aberrantly O-glycosylated polymeric immunoglobulin A1 (IgA1), i.e., galactose-deficient IgA1 (Gd-IgA1), as the pivotal component at the beginning of IgAN pathogenesis (10, 11), are associated with a poor prognosis in IgAN (11). Staining for Gd-IgA1 was relatively specific in the glomeruli of patients with IgAN (12). Gd-IgA1 combined with the glycan-specific immunoglobulin G (IgG) or immunoglobulin A (IgA) autoantibodies, (13) and formed of Gd-IgA1–IgG/IgA immune complexes, deposited in the glomeruli and then induced the activation of the complement system and an inflammation reaction (14). The components of complement activation are usually detected in both the circulation and renal biopsy tissues of IgAN (15). Complement component 3 (C3) accompanies IgA deposited in the renal tissue of approximately 90% patients with IgAN (16). In recent years, increasing evidence has confirmed the important role of abnormal complement activation in the pathogenesis of IgAN (15), whereas Gd-IgA1 and abnormal activation of the complement system are rarely observed in MCD. It is unclear whether or not the IgA deposited in MCD-IgAN is the same pathogenic Gd-IgA1 as in general IgAN, and whether or not the degree of complement system activation in MCD-IgAN is the same as in general IgAN.

In this study, we explored the role of Gd-IgA1 and complement C3 in IgAN with MCD (MCD-IgAN) and IgAN without MCD (non-MCD-IgAN). We aimed to determine whether unusual cases of MCD-IgAN are a special clinical subtype of IgAN or MCD accompanied by coincidental IgA deposition.

## Material and methods

### Ethics statement

This research was conducted according to the principles of the Declaration of Helsinki. The ethics committees of Beijing Anzhen Hospital approved the study and all enrolled individuals provided informed consent to participate in this investigation.

### Study participants

**Figure 1:**
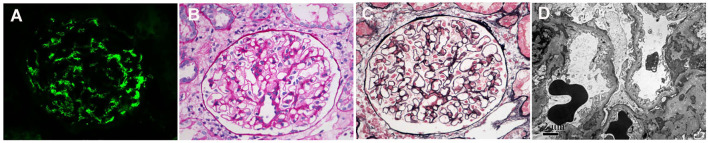
Immunofluorescence, light microscopy, and electron microscopy in patients with minimal change disease (MCD)-immunoglobulin A nephropathy (IgAN). **(A)** Immunofluorescence of immunoglobulin A (IgA). **(B)** Periodic acid–silver methenamine. **(C)** Masson’s trichrome (PAM–Masson) staining. **(D)** diffuse effacement of podocyte foot processes under electron microscopy. **(A–C)**: original magnification ×400; **(D)**: original magnification ×8000.

This was a retrospective study of patients with IgAN in Beijing Anzhen Hospital. A total of 926 patients were diagnosed with primary IgAN from January 2010 to September 2022. Among them were 51 patients who had unremarkable glomeruli under light microscopy (only one patient had mild mesangial hypercellularity; see **Table 1**) and diffuse (over 80%) foot process effacement under electron microscopy (**Figure 1**). Because these findings, other than the mesangial IgA deposition, indicate MCD, this group was defined as the MCD-IgAN group. In addition, 51 patients with IgAN without MCD (i.e., the non-MCD-IgAN group), from June 2019 to February 2020, were consecutively enrolled as the disease control.

**Table 1:** Demographic, clinical, and histologic characteristics of patients with minimal change disease (MCD)-immunoglobulin A nephropathy (IgAN) and non-MCD-IgAN.

Characteristic	Mean ± SD or median (IQR)		*p*-value
	MCD-IgAN (*N* = 51)	Non-MCD-IgAN (*N* = 51)	
Clinical features			
Male/female (n)	39/12	19/32	< 0.001
Age (years)	36.1 ± 16.1	40.47 ± 14.2	0.147
HBP, *n* (%)	12 (25.5)	30 (58.8)	0.001
SBP (mmHg)	120 (115–130)	129 (118–141)	0.017
DBP (mmHg)	80 (70–85)	82 (75–94)	0.095
Prodromic infection, *n* (%)	1 (2.1)	7 (13.7)	0.034
Gross hematuria, *n* (%)	0 (0.0)	7 (13.7)	0.008
Microscopic hematuria, *n* (%)	10 (19.6)	45 (88.2)	< 0.001
Laboratory measurements			
Initial proteinuria (g/day)	7.32 (3.70–12.38)	1.17 (0.60–2.61)	< 0.001
Albumin (g/L)	21.05 ± 6.84	41.12 ± 5.96	< 0.001
Serum creatinine (μmol/l)	77.5 (62.0–93.0)	76.7 (61.3–102.8)	0.995
eGFR (mL/min/1.73 m^2^)	102.9 (88.1–124.8)	96.21 (56.40–119.55)	0.086
Uric acid (μmol/L)	375.0 (315.0–437.5)	355.8 (278.8–477.3)	0.345
Serum IgA (g/L)	2.94 ± 1.22	3.08 ± 1.11	0.528
Plasma C3 (g/L)	1.35 ± 0.27	1.16 ± 0.23	< 0.001
Plasma C4 (g/L)	0.34 ± 0.11	0.27 ± 0.09	0.003
Serum TG (mmol/L)	2.30 (1.65–2.85)	1.77 (0.90–2.62)	0.013
Serum TCHO (mmol/L)	9.60 (7.62–12.65)	5.13 (4.14–6.19)	< 0.001
Serum LDL-C (mmol/L)	6.19 (4.85–9.14)	3.09 (2.48–3.81)	< 0.001
Serum Gd-IgA1 (μg/mL)	4.61 (2.83–6.38)	7.44 (4.87–10.32)	0.001
Histologic features			
Glomerular IgA deposits (2+, 3+, 4+), *n* (%)	46 (90.2), 5 (9.8), 0 (0.0)	19 (37.3), 31 (60.8), 1 (2.0)	< 0.001
Glomerular C3 deposits (0, 1+, 2+, 3+ and 4+), *n* (%)	13 (25.5), 16 (31.4), 18 (35.3), 4 (7.8)	2 (3.9), 2 (3.9), 30 (58.8), 17 (33.3)	< 0.001
Glomerular Gd-IgA1 deposits (0, 1+, 2+, 3+/4+), *n* (%)	44 (86.3), 4 (7.8), 2 (3.9), 1 (2.0)	9 (17.6), 19 (37.3), 18 (35.3), 5 (9.8)	< 0.001
Oxford classification, *n* (%)			
M1	1 (2.0)	22 (43.1)	< 0.001
E1	0 (0.0)	27 (52.9)	< 0.001
S1	0 (0.0)	17 (33.3)	< 0.001
T1, T2	0 (0.0), 0 (0.0)	16 (31.4), 8 (15.7)	< 0.001
C1, C2	0 (0.0), 0 (0.0)	16 (31.4), 4 (7.8)	< 0.001

IgAN, immunoglobulin A nephropathy; MCD, minimal change disease; IQR, interquartile range; SD, standard deviation; HBP: high blood pressure; SPB: systolic blood pressure; DBP: diastolic blood pressure; eGFR, estimated glomerular filtration rate; IgA, immunoglobulin A; C3, complement C3; C4, complement C4; TG, triglyceride; TCHO, total cholesterol; LDL-C, low-density lipoprotein cholesterol; Gd-IgA1, galactose-deficient immunoglobulin A1.

Oxford classifications: mesangial hypercellularity score (M1 > 0.5); the presence of endocapillary proliferation (E1: present); segmental glomerulosclerosis/adhesion (S1: present); severity of tubular atrophy/interstitial fibrosis (T1: 26%–50%, T2: > 50%); and presence of crescent (C1: 1%–25%, C2: 26%–100%).

### Clinical and histologic characteristics

The clinical characteristics of recruited patients at the time of renal biopsy were collected from the medical records. These included age, gender, history of high blood pressure [i.e., a systolic blood pressure (SBP) of 140 mmHg or more, a diastolic blood pressure (DSP) of 90 mmHg or more, or taking antihypertensive medication to prevent hypertension], prodromic infection, gross hematuria, microscopic hematuria, 24-hour urine protein excretion, serum creatinine levels, and plasma C3 and C4 levels. Microscopic hematuria was defined as more than three red blood cells per high-power field under light microscopic examination of sediment after centrifugation. The estimated glomerular filtration rate (GFR) was calculated using the modified GFR estimating equation (17). Histologically, the Oxford classification was used to evaluate the pathological lesions for those with more than eight glomeruli in biopsy specimens (18).

### Detection of glomerular Gd-IgA1 by immunofluorescence in renal biopsies

The paraffin-embedded biopsy renal tissues were cut into slices of 4 μm thickness, deparaffinized, and incubated with pepsase for 35 minutes at 37°C. After being washed with phosphate-buffered saline (PBS), sections were incubated in 3% bovine serum albumin (BSA; Sigma-Aldrich, St. Louis, MO, USA) blocking solution for 30 minutes at 25°C. Anti-human Gd-IgA1 antibody, produced by clone KM55 (Immuno-Biological Laboratories, Fujioka, Japan), with a dilution of 1:200 in PBS were incubated overnight at 4°C. After being washed, Cy™3-conjugated AffiniPure donkey anti-rat antibodies (diluted 1:200 in PBS; Jackson ImmunoResearch Laboratories, Philadelphia, PA, USA) were added for 1 hour at 37°C. After being washed with PBS three times for 5 minutes each, the sections were air dried in the dark and mounted with mounting medium containing 4′,6-diamidino-2-phenylindole (DAPI).

The methods to detect colocalization of Gd-IgA1 and IgA1, and Gd-IgA1 and C3c were as follows. The paraffin-embedded biopsy renal tissues were cut into slices of 4 μm thickness, deparaffinized, and subjected to antigen retrieval and BSA blocking. Gd-IgA1 produced by clone KM55 (Immuno-Biological Laboratories) and Cy™3-conjugated AffiniPure donkey anti-rat antibodies (diluted 1:200 in PBS; Jackson ImmunoResearch Laboratories) were added as above, one after the other. Next, fluorescein isothiocyanate (FITC)-conjugated rabbit anti-human IgA (Dako, Glostrup, Denmark) and FITC-conjugated rabbit anti-human C3c (Dako) IgA was added to Gd-IgA1, and C3c was added to Gd-IgA1, respectively (19).

Primary antibodies were replaced by PBS as blank controls. = Glomerular immunofluorescence was scored using fluorescence microscopy (Nikon 80i, Japan). The staining intensity of anonymized renal biopsies as negative: 0 (absent), positive: 1+, 2+, 3+ and 4+ were grades by two observers (Wy G and Lj S), who were blinded to clinical data. We excluded sections that contained fewer than two glomeruli.

A two-dimensional (2D) fluorogram obtained using Image-Pro Plus software was used to analyze the fluorescence colocalization. Pearson’s correlation and overlap coefficient were used to quantify the degree of colocalization (19).

### Detection of serum Gd-IgA1 by enzyme-linked immunosorbent assay

Serum samples from 37 patients with MCD-IgAN and 48 patients with non-MCD-IgAN were collected on the morning of biopsy, divided into aliquots, and stored at –80°C until the measurement of circulation Gd-IgA1. Serum was also collected from 54 age-, gender-, and geographically matched healthy adults selected as healthy controls. The levels of serum Gd-IgA1 were measured using a commercially available enzyme-linked immunosorbent assay (ELISA) test kit with KM55 (Immuno-Biological Laboratories, Fujioka, Japan), in accordance with the manufacturer’s protocol.

### Statistical analysis

Quantitative variables were summarized as the mean ± standard deviation (for normally distributed data), or median and interquartile range (IQR) (for non-normally distributed data). Categorical variables were expressed as the number and percentage. The Kolmogorov–Smirnov test was used to analyze the normality of the distribution of variables. For continuous variables, if the data were normally distributed, an independent-samples *t*-test was used; if not, a Mann–Whitney *U*-test or Kruskal–Wallis *H*-test was performed. For categorical variables, a chi-squared test was used. The statistical software Statistical Product and Service Solutions (SPSS; IBM SPSS Statistics), version 23.0 (IBM Corporation, Armonk, NY, USA), was used for the analysis. Statistical significance was set at a *p*-value of less than 0.05.

## Results

### Association of clinical, laboratory, and pathologic parameters in patients in the MCD-IgAN group and those in the non-MCD-IgAN group

The clinical, laboratory, and histologic features of the 51 recruited patients with IgAN are summarized in **Table 1**. A total of 39 (76.5%) IgAN patients were male, and they had a mean age of 36.1 ± 16.1 years at the time of the renal biopsy. There were significantly fewer patients in the MCD-IgAN group than in the non-MCD-IgAN group who experienced hypertension [12/51 (23.5%) vs. 30/51 (58.8%), *p* < 0.001; see **Table 1**], microscopic hematuria [1.96% (19.6%) vs. 45/51 (88.2%), *p* < 0.001; see **Table 1**], and prodromic infection [1/51 (2.1%) vs. 7/51 (13.7%), *p* = 0.034; see **Table 1**]. In addition, compared with those in the non-MCD-IgAN group, patients in the MCD-IgAN group presented with more initial proteinuria [7.32 g/day (3.70–12.38 g/day) vs. 1.17 g/day (0.60–2.61 g/day), *p* < 0.001; see **Table 1**] and lower levels of serum albumin (21.05 ± 6.84 g/L vs. 41.12 ± 5.96 g/L, *p* < 0.001; see **Table 1**). There was no significant difference between the MCD-IgAN group and the non-MCD-IgAN group in the level of serum creatinine [77.5 μmol/L (62.0–93.0 μmol/L) vs. 76.7 μmol/L (61.3–102.8 μmol/L), *p*= 0.995; see **Table 1**] and estimated glomerular filtration rate (eGFR) [102.9 mL/min/1.73 m^2^ (88.1–124.8 mL/min/1.73 m^2^) vs. 96.2 mL/min/1.73 m^2^ (56.4–119.6 mL/min/1.73 m^2^), *p* = 0.086; see **Table 1**]. Histologically, fewer patients in the MCD-IgAN group than in the non-MCD-IgAN group presented with mesangial hypercellularity (M1) [1 (2%) vs. 22 (43.1%), *p* < 0.001; see **Table 1**].

### Association of clinical, laboratory, and pathologic parameters in the Gd-IgA1-positive subgroup and the Gd-IgA1-negative subgroup in patients with IgAN

In the MCD-IgAN group, glomerular Gd-IgA1-positive patients were, on average, younger than glomerular Gd-IgA1-negative patients (25.0 ± 8.4 years vs. 38.0 ± 16.5 years, *p* = 0.006). There was a significant difference between the Gd-IgA1-positive subgroup and Gd-IgA1-negative subgroup in the initial levels of proteinuria [7.75 g/day (3.73–14.85 g/day) vs. 7.32 g/day (3.70–11.70 g/day), *p* = 0.707], serum albumin (18.68 ± 4.68 g/L vs. 21.49 ± 7.13 g/L, *p* = 0.290), serum creatinine [81.5 μmol/L (56.0–93.0 μmol/L) vs. 77.0 μmol/L (62.0–88.0 μmol/L), *p* = 849], eGFR [101.5 mL/min/1.73 m^2^ (98.4–125.5 mL/min/1.73 m^2^) vs. 103.4 mL/min/1.73 m^2^ (87.3–124.8 mL/min/1.73 m^2^), *p* = 0.694], serum IgA (2.96 ± 0.94 g/L vs. 2.93 ± 1.28 g/L, *p* = 0.945), and serum complement C3 (1.35 ± 0.34 g/L vs. 131 ± 0.24 g/L, *p* = 0.920) (**Table 2**).

**Table 2:** Demographic, clinical, and histologic characteristics of patients with immunoglobulin A nephropathy based on the staining of galactose-deficient immunoglobulin A1 in renal tissues.

Characteristic	MCD-IgAN		non-MCD-IgAN		*p-value	**p-value	***p-value
	Gd-IgA1 positive (*N* = 8)	Gd-IgA1 negative (*N* = 43)	Gd-IgA1 positive (*N* = 42)	Gd-IgA1 negative (*N* = 9)			
Clinical features							
Male/female (*n*)	6/2	33/10	16/26	3/6	0.915	0.789	0.054
Age (years)	25.9 ± 8.4	38.0 ± 16.5	39.0 ± 13.8	47.3 ± 15.0	0.006	0.111	0.003
HBP, *n* (%)	0 (0.0)	13 (30.2)	23 (54.8)	7 (77.8)	0.023	0.203	0.004
SBP (mmHg)	119 (108–120)	120 (115–135)	129 (118–140)	133 (122–169)	0.125	0.277	0.22
DBP (mmHg)	78 (70–80)	80 (70–86)	83 (74–94)	78 (75–91)	0.223	0.688	0.50
Prodromic infection, *n* (%)	0 (0.0)	1 (2.5)	6 (14.3)	1 (11.1)	0.651	0.802	0.254
Microscopic hematuria, *n* (%)	0 (0.0)	10 (23.3)	39 (92.9)	6 (66.7)	0.128	0.027	< 0.001
Laboratory measurements							
Initial proteinuria (g/day)	7.75 (3.73–14.85)	7.32 (3.70–11.70)	0.95 (0.56–2.29)	2.71 (0.78–4.25)	0.707	0.035	< 0.001
Albumin (g/L)	18.68 ± 4.68	21.49 ± 7.13	41.77 ± 4.72	38.06 ± 9.76	0.290	0.294	< 0.001
Serum creatinine (μmol/l)	81.5 (56.0–93.0)	77.0 (62.0–88.0)	76.6 (62.5–105.7)	76.7 (51.6–99.2)	0.849	0.503	0.907
eGFR (mL/min/1.73 m^2^)	101.5 (98.4–125.5)	103.4 (87.3–124.8)	94.7 (56.3–120.0)	106.4 (53.7–118.7)	0.694	0.725	0.168
Uric acid (μmol/L)	394.7 (315.4–442.3)	374.0 (315.0–428.0)	346.7 (289.8–453.9)	357.9 (261.2–503.7)	0.720	0.875	0.559
Serum IgA (g/L)	2.96 ± 0.94	2.93 ± 1.28	3.14 ± 1.17	2.82 ± 0.78	0.945	0.436	0.687
Plasma C3 (g/L)	1.35 ± 0.34	1.31 ± 0.24	1.12 ± 0.23	1.33 ± 0.18	0.920	0.013	< 0.001
Plasma C4 (g/L)	0.38 ± 0.08	0.34 ± 0.11	0.28 ± 0.08	0.30 ± 0.12	0.417	0.491	0.006
Serum TG (mmol/L)	3.14 (1.41–4.28)	2.29 (1.65–2.73)	1.55 (0.85–2.31)	2.14 (1.51–3.85)	0.174	0.124	0.017
Serum TCHO (mmol/L)	11.94 (6.14–13.48)	9.33 (7.62–12.56)	4.94 (4.06–5.83)	6.20 (5.22–7.36)	0.749	0.016	0.002
Serum LDL-C (mmol/L)	7.65 (3.85–10.27)	6.14 (4.85–8.82)	3.07 (2.44–3.58)	3.81 (2.56–5.11)	0.495	0.144	0.001
Serum Gd-IgA1 (μg/mL)	3.26 (3.13–4.71)	4.83 (2.55–6.44)	8.42 (5.01–10.32)	5.83 (2.28–11.63)	0.531	0.333	0.008
Histologic features							
Glomerular IgA deposits (2+, 3+, 4+), *n* (%)	5 (62.5), 3 (37.5), 0 (0.0)	41 (95.3), 2 (4.7), 0 (0.0)	12 (28.6), 29 (69.0), 1 (2.4)	7 (77.8), 2 (22.2), 0 (0.0)	0.004	0.007	0.066
Glomerular C3 deposits (0, 1+, 2+, 3+/4+), *n* (%)	2 (25.0), 2 (25.0), 3 (37.5), 1 (12.5)	11 (25.6), 14 (32.6), 15 (34.9), 3 (7.0)	0 (0.0), 1 (2.4), 26 (61.9), 15 (35.7)	2 (22.2), 1 (11.1), 4 (44.4), 2 (22.2)	0.692	0.10	0.001
Oxford classification, *n* (%)							
M1	0 (0.0)	1 (2.3)	20 (47.6)	2 (22.2)	0.663	0.163	0.002
E1	0 (0.0)	0 (0.0)	26 (61.9)	1 (11.1)	–	0.006	0.001
S1	0 (0.0)	0 (0.0)	15 (35.7)	2 (22.2)	–	0.436	0.046
T1, T2	0 (0.0), 0 (0.0)	0 (0.0), 0 (0.0)	15 (35.7), 6 (14.3)	1 (11.1), 2 (22.2)	–	0.347	0.018
C1, C2	0 (0.0), 0 (0.0)	0 (0.0), 0 (0.0)	16 (38.1), 4 (9.5)	0 (0.0), 0 (0.0)	–	0.016	0.022

IgAN, immunoglobulin A nephropathy; MCD, minimal change disease; HBP, high blood pressure; SPB, systolic blood pressure; DBP, diastolic blood pressure; eGFR, estimated glomerular filtration rate; C3, complement C3; C4, complement C4; TG, triglyceride; TCHO, total cholesterol; LDL-C, low density lipoprotein cholesterol; Gd-IgA1, galactose-deficient immunoglobulin A1; IgA, immunoglobulin A.

Oxford classifications: mesangial hypercellularity score (M1 > 0.5); the presence of endocapillary proliferation (E1: present); segmental glomerulosclerosis/adhesion (S1: present); severity of tubular atrophy/interstitial fibrosis (T1: 26%–50%, T2 >50%); and presence of crescent (C1: 1%–25%, C2: 26%–100%).

*p-value was used to indicate the difference between the Gd-IgA1-positive subgroup and Gd-IgA1-negative subgroup in patients with MCD-IgAN; **p-value was used to indicate the difference between the Gd-IgA1-positive subgroup and Gd-IgA1-negative subgroup in patients with non-MCD-IgAN; and ***p-value was used to indicate the difference between the MCD-IgAN Gd-IgA1-positive subgroup and non-MCD-IgAN Gd-IgA1-positive subgroup.

A two-tailed p-value < 0.05 was considered to be statistically significant.

In the non-MCD-IgAN group, more patients in the glomerular Gd-IgA1-positive subgroup presented with microscopic hematuria [39 (92.9%) vs. 6 (66.7%), *p* = 0.027]. In addition, patients in the Gd-IgA1-positive subgroup showed lower levels of initial proteinuria [0.95 g/day (0.56–2.29 g/day) vs. 2.71 g/day (0.78–4.25 g/day), *p* = 0.035] and complement C3 (1.12 ± 0.23 g/L vs. 1.33 ± 0.18 g/L, *p* = 0.013) than the Gd-IgA1-negative subgroup. There was a significant difference between the Gd-IgA1-positive subgroup and the Gd-IgA1-negative subgroup in levels of serum albumin (41.77 ± 4.72 g/L vs. 38.06 ± 9.76 g/L, *p* = 0.294) and eGFR (94.7 mL/min/1.73 m^2^ (56.3–120.0 mL/min/1.73 m^2^) vs. 106.4 mL/min/1.73 m^2^ (53.7–118.7 mL/min/1.73 m^2^), *p* = 0.725). Histologically, more patients in the Gd-IgA1-positive subgroup presented with severe histologic lesions than in the Gd-IgA1-negative subgroup: S1 [15 (35.7%) vs. 2 (22.2%), *p* = 0.006; and C1/C2 [16 (38.1%)/4 (9.5%) vs. 0 (0.0%)/0 (0.0%), *p* = 0.016; see **Table 2**].

### Gd-IgA1 in serum and deposits in renaltissues between patients in the MCD-IgAN group and non-MCD-IgAN group

**Figure 2:**
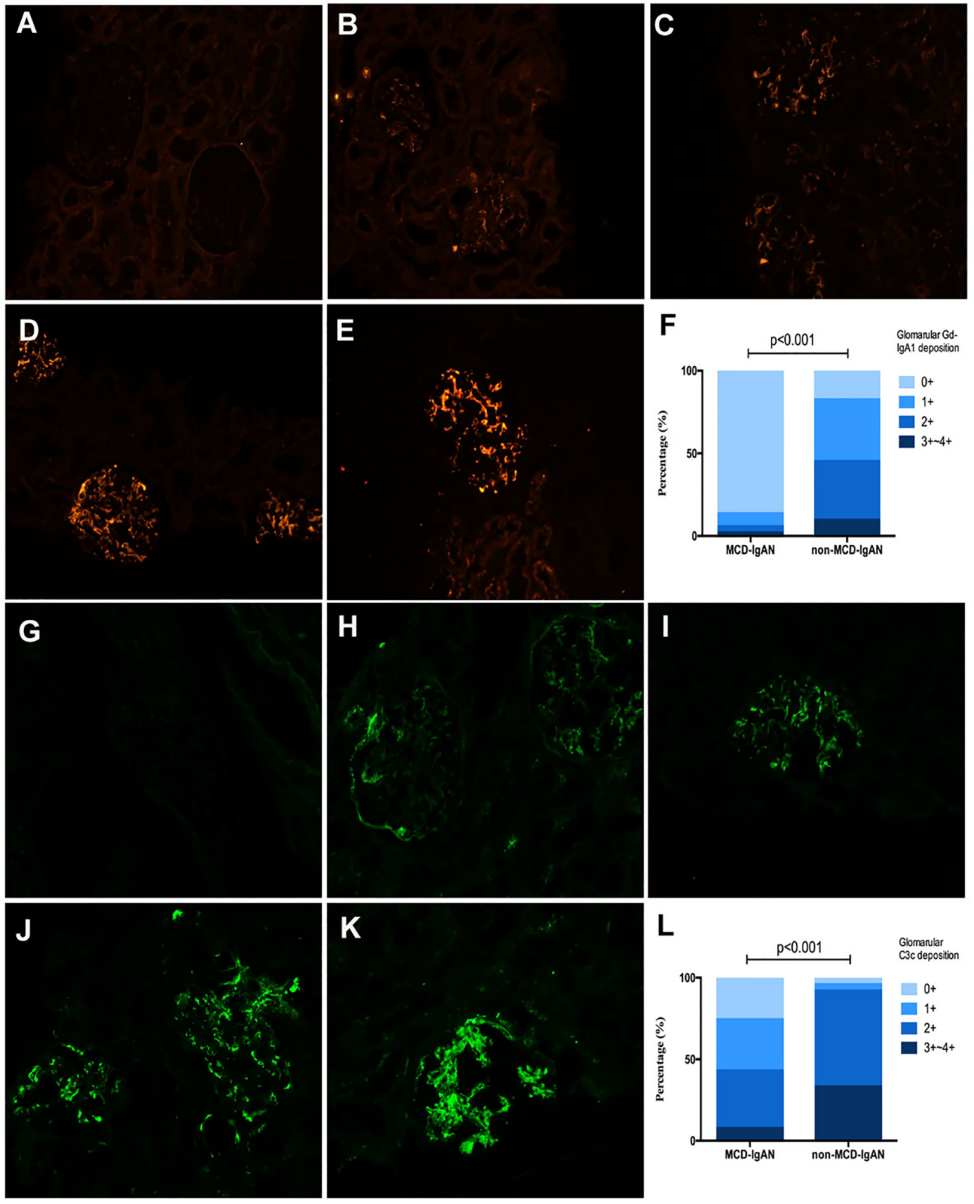
Representative pictures of immunofluorescence staining of mesangial galactose-deficient immunoglobulin A1 (Gd-IgA1) and C3c 0 to 4+ in patients with immunoglobulin A nephropathy (IgAN). Granular-positive staining of Gd-IgA1 **(A–E)** and C3c **(G–K)** by immunofluorescencealong the glomerular mesangial and capillary area in patients with IgAN. **(A, G)** negative; **(B, H)** 1+ intensity; **(C, I)** 2+ intensity; **(D, J)** 3+ intensity, and **(E, K)** 4+ intensity. **(A–E, G–K)**: original magnification ×200. **(F)** The intensity of Gd-IgA1 deposition in the glomerular mesangial and capillary areas was weaker in the minimal change disease (MCD)-IgAN group than in the non-MCD-IgAN group. **(L)** The intensity of C3c deposition in the glomerularmesangial and capillary areas was also weaker in MCD-IgAN group than in the non-MCD-IgAN group.

**Figure 3:**
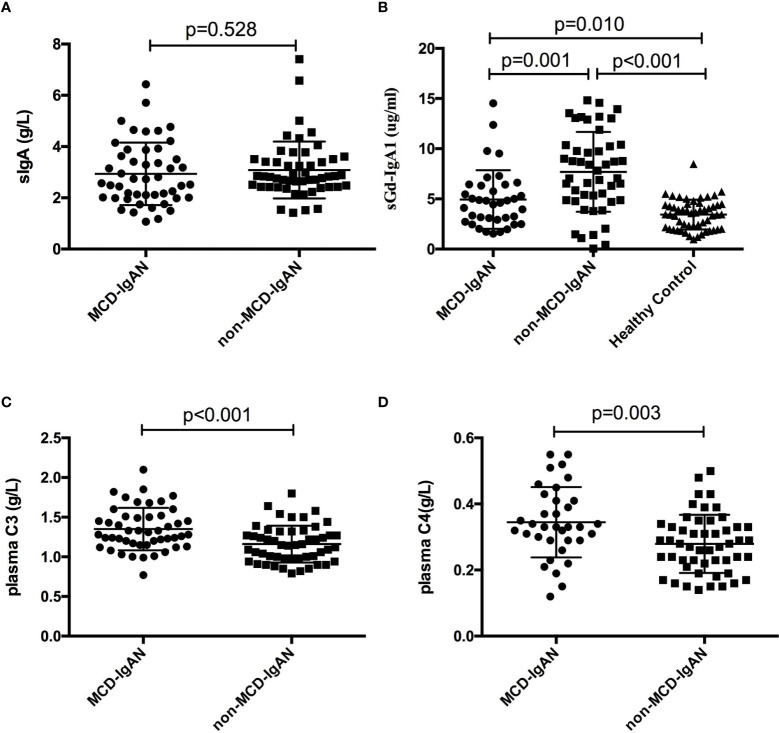
Levels of serum galactose-deficient immunoglobulin A1 (Gd-IgA1) and immunoglobulin A (IgA), plasma component 3 (C3) and C4 in the minimal change disease (MCD)-immunoglobulin A nephropathy (IgAN) group and non-MCD-IgAN group. **(A)** There was no difference between the MCD-IgAN group and non-MCD-IgAN group in the level of serum immunoglobulin (IgA). **(B)** The level of serum Gd-IgA1 in the MCD-IgAN group was lower than in the non-MCD-IgAN group, but higher than in the healthy control participants. The levels of plasma complement C3 **(C)** and complement C4 **(D)** were higher in the MCD-IgAN group than in the MCD-IgAN group.

Gd-IgA1 deposited in the mesangial and capillary areas in 7 out of 51 (13.7%) patients with MCD-IgAN and in 42 out of 51 (82.4%) patients with non-MCD-IgAN. Granular-positive staining of Gd-IgA1 by immunofluorescence was along the glomerular mesangial and capillary area in patients with IgAN (**Figure 2A–E**). In histologic features, patients in the MCD-IgAN group presented with lower mesangial Gd-IgA1 deposition [0/1+/2+/3 and 4+: 44 (86.3%)/4 (7.8%)/2 (3.9%)/1 (2.0%) vs. 9 (17.6%)/19 (37.3%)/18 (35.5%)/5 (9.8%), *p* < 0.001; see **Table 1**] and IgA deposition [2+/3+/4+: 46 (90.2%)/5 (9.8%)/0 (0.0%) vs. 19 (37.3%)/31 (60.8%)/1 (2.0%), *p* < 0.001; see **Table 1**, **Figure 2F**] in glomeruli than those in the non-MCD-IgAN group. There was no significant difference in levels of serum IgA between patients in the MCD-IgAN group and those in non-MCD-IgAN group (2.94 ± 1.22 g/L vs. 3.08 ± 1.11 g/L, *p* = 0.528; see **Table 1**, **Figure 3A**). Moreover, the level of serum Gd-IgA1 in patients with MCD-IgAN was lower than in patients with non-MCD-IgAN [4.61 μg/mL (2.83–6.38 μg/mL) vs. 7.44 μg/mL (4.87–10.32 μg/mL), *p* = 0.001; see **Table 1**, **Figure 3B**] and was higher in the MCD-IgAN group than in the healthy control group [4.61 μg/mL (2.83–6.38 μg/mL) vs. 3.45 μg/mL (2.01–4.42 μg/mL), *p* = 0.010; see **Table 1**, **Figure 3B**].

### Plasma C3 and glomerular C3c in patients in the MCD-IgAN group and those in the non-MCD-IgAN group

Plasma levels of C3 were higher in patients with MCD-IgAN than in those with non-MCD-IgAN (1.35 ± 0.27 g/L vs. 1.16 ± 0.23 g/L, *p* < 0.001; see **Table 1**, **Figure 3C**). In addition, plasma levels of C4 were higher in patients with MCD-IgAN than in patients with non-MCD-IgAN (0.34 ± 0.11 g/L vs. 0.27 ± 0.09 g/L, *p* = 0.003; see **Table 1**, **Figure 3D**). Patients in the MCD-IgAN group exhibited a lower intensity of glomerular C3c than those in IgAN-non-group [0/1+/2+/3 and 4+: 13 (25.5%)/16 (31.4%)/18 (35.3%)/4 (7.8%) vs. 2 (3.9%)/2 (3.9%)/30 (58.8%)/17 (33.3%), *p* < 0.001; see **Table 1**, **Figures 2G–L**].

### Colocalization of Gd-IgA1 and IgA, and of Gd-IgA1 and C3c, along the glomerular mesangial areas in patients with IgAN

**Figure 4:**
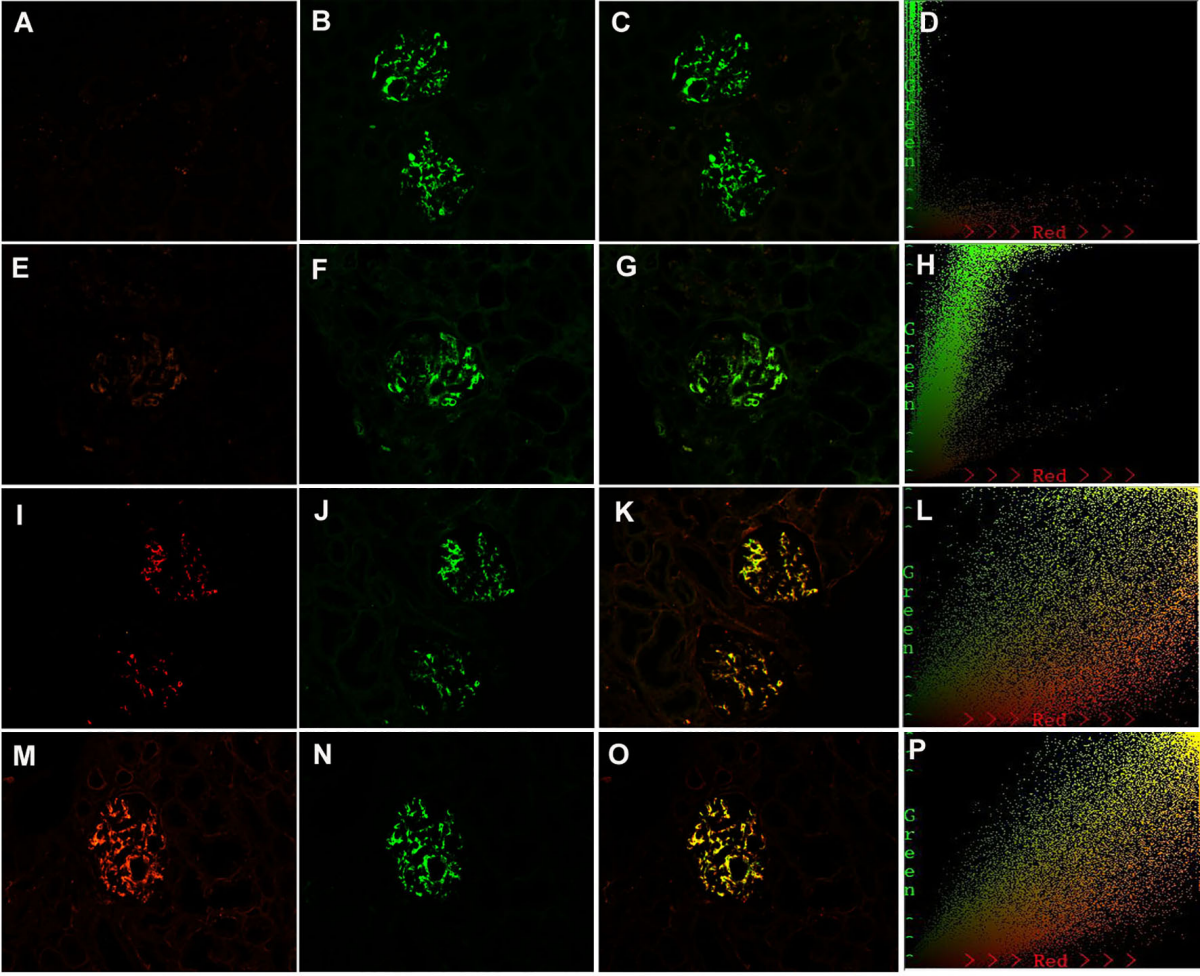
Paraffin-embedded sections for of galactose-deficient immunoglobulin A1 (Gd-IgA1) and immunoglobulin A (IgA) by immunofluorescence in patients with immunoglobulin A nephropathy (IgAN). Granular-positive staining of Gd-IgA1 negative **(A)**, 1+ **(E)**, 2+ **(I)**, 3+ **(H)** by immunofluorescence along the glomerular mesangial and capillary area in the same section with IgA. Granular-positive staining of IgA **(B, F, J, N)** by immunofluorescence along the glomerular mesangial and capillary area in patients with IgAN. **(G, K, O)** Gd-IgA1 and IgA colocalized completely along the glomerular mesangial and capillary area. The corresponding two-dimensional (2D) fluorograms have been included to confirm the degree of colocalization. (H: Pearson’s correlation = 0.890362, overlap coefficient = 0.902370; **(L)**: Pearson’s correlation = 0.851353, overlap coefficient = 0.855853; **(P)**: Pearson’s correlation = 0.928907, overlap coefficient = 0.925787). **(A–C)**, **(E–G)**, **(I–K)**, and **(M–O)**: original magnification ×200.

As 7 out of 51 (13.7%) patients in the MCD-IgAN group and 42 out of 51 (82.4%) patients in the non-MCD-IgAN group presented with glomerular mesangial Gd-IgA1 deposition, we further investigated the colocalization of IgA and Gd-IgA1 (**Figure 4**). Gd-IgA1 and IgA colocalized along the glomerular mesangial areas in both the MCD-IgAN group and the non-MCD-IgAN group (**Figure 4**).

**Figure 5:**
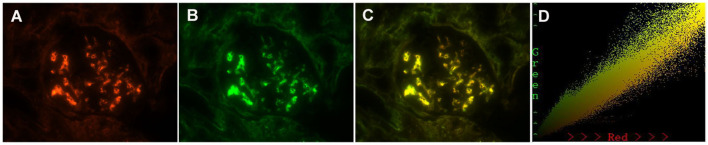
Paraffin-embedded sections for colocalization of galactose-deficient immunoglobulin A1 (Gd-IgA1) and C3c by immunofluorescence in patients with immunoglobulin A nephropathy (IgAN). Granular-positive staining of Gd-IgA1 **(A)** by immunofluorescence along the glomerular mesangial area in patients with IgAN. Granular-positive staining of C3c **(B)** by immunofluorescence along the glomerular mesangial area in the same section with Gd-IgA1. **(C)** Gd-IgA1 and C3c colocalized completely along the glomerular mesangial and capillary area. **(D)** The corresponding two-dimensional (2D) fluorograms have been included to confirm the degree of co-localization (Pearson’s correlation = 0.976378, overlap coefficient = 0.985598). **(A–C)**: original magnification ×400.

In the non-MCD-IgAN group, glomerular Gd-IgA1, KM55, colocalization was strongly correlated with renal local complement activation (glomerular C3c deposition) [glomerular Gd-IgA1 0/1+/2+/3 and 4+: C3c 0: 2 (100.0%)/0 (0.0%)/0 (0.0%)/0 (0.0%) vs. C3c 1+: 1 (50.0%)/1 (50.0%)/0 (0.0%)/0 (0.0%) vs. C3c 2+: 4 (13.3%)/13 (43.3%)/10 (33.3%)/3 (10.0%) vs. 2 (11.8%)/5 (29.4%)/8 (47.1%)/2 (11.8%), *p* = 0.014; see **Table 2**]. We also investigated the colocalization of Gd-IgA1 and C3c in patients with IgAN by immunofluorescence. Gd-IgA1 and C3c colocalized along the glomerular mesangium in patients with IgAN (**Figures 5A–C**). Two-dimensional (2D) fluorograms were used to quantify the colocalization and confirmed the degree of colocalization (**Figure 5D**; Pearson’s correlation = 0.976378; overlap coefficient = 0.985598).

## Discussion

Patients with IgAN display various clinical manifestations and pathological phenotypes. Approximately 5%–10% of patients with IgAN exhibit onset nephrotic syndrome (5, 20), with histologic features of mild mesangial lesions, by light microscopy, and diffuse foot process effacement of podocytes, by electron microscopy, representative of MCD, which is defined as MCD-IgAN. The specific pathogenesis of MCD-IgAN is still not entirely clear (3, 9). In this study, we found not only that the clinical and histologic features of MCD-IgAN were different from those of classic IgAN, but also that the underlying mechanisms may differ from IgAN.

We found that the clinical manifestation of MCD-IgAN greatly differed from that of IgAN, as previous reported (21). IgAN usually presented with recurrent episodes of gross hematuria after mucosal infections, and with subnephrotic proteinuria. Conversely, MCD-IgAN usually presented with nephrotic proteinuria and hypoproteinemia, and seldom hematuria, which more closely resembles MCD (21–25). Previous studies also revealed that there are no significant differences in clinical manifestation and prognosis between MCD-IgAN and MCD (22). In terms of therapy, patients with MCD-IgAN presented better renal outcomes than patients with non-MCD-IgAN (21). Patients with MCD-IgAN usually achieve a comparable clinical outcome to those with MCD, requiring corticosteroid and immunosuppressive medication in addition to the treatments used for MCD (23). However, some studies have reported that patients with MCD-IgAN respond well and safely to corticosteroid therapy (24, 25), MCD-IgAN experience a higher recurrence rate than non-MCD-IgAN. (25) IgAN and MCD may also coexist in children, and most of these patients who presented with nephrotic syndrome responded well to corticosteroids and had a satisfying prognosis (26).

The pathophysiology of IgAN is considered to be a multi-hit mechanism (9). Gd-IgA1 is the key component in the pathogenesis of IgAN (10). In this study, we found that, compared with non-MCD-IgAN patients, MCD-IgAN patients presented with lower levels of serum Gd-IgA1, which was consistent with previous research (6). However, the levels of Gd-IgA1 were much higher in patients with MCD-IgAN than in healthy control participants. Furthermore, unlike previous studies (6, 22), we found that Gd-IgA1 was rarely deposited in the glomerulus of patients with MCD-IgAN. Gd-IgA1 was deposited in the glomerulus in only 13.7% of patients with MCD-IgAN, compared with 82.4% of patients with non-MCD-IgAN. Our study suggested that most IgA deposited in this variant form of IgAN was normal IgA but not Gd-IgA1. A previous study showed that, compared with poly-IgA1 (pIgA1) complexes from non-MCD-IgAN, pIgA1 complexes from MCD-IgAN could induce weaker effects on mesangial inflammatory cytokine production (6). These findings may suggest that the IgA deposited in patients with MCD-IgAN was not as pathophysiological as that deposited in patients in IgAN. The presence of incidental IgA deposition in donor kidneys (from individuals with no known underlying kidney disease) appears to be quite high in Asian countries, particularly in China and Japan (27, 28). Unfortunately, we do not have information about the incidence of glomerular IgA depositions in donor kidney biopsies in our study hospital.

Interestingly, we found that the activation of the complement system was more severe in non-MCD-IgAN patients than in MCD-IgAN patients. Compared with patients with MCD-IgAN, plasma levels of C3 and C4 were significantly lower in patients with non-MCD-IgAN. Moreover, the intensity of C3c deposition in MCD-IgAN patients was weaker than in non-MCD-IgAN patients. Increasing evidence has implied that alternative pathway- and lectin pathway-induced complement activation has an important role in the pathogenesis of IgAN (15, 29, 30). In three different pathways. These three pathways converge at the C3 level and lead to the formation of C3 convertase, inducing the cleavage of C3, and then inducing the activation of the common terminal pathway and triggering the formation of C5b-9 (15). In patients with IgAN, the activation of the complement system occurs both in the systemic circulation and in the renal locality. The intensity of renal local C3c deposition and the level of plasma C3 could represent the degree of complement activation. Overactivation of systemic complement results in C3 consumption and, thus, low plasma C3 levels, and renal local complement activation leads to greater C3c deposition in glomeruli (31). A previous study showed that lower levels of C3 and stronger intensity of C3 deposition in glomeruli led to poor renal outcomes in patients with IgAN (32). In this study, we found that the activation of the complement system, both in the circulation and in the renal locality, was much weaker in patients with MCD-IgAN than in those with non-MCD-IgAN.

In conclusion, our study suggests that most IgA that was deposited in MCD-IgAN was not as pathogenic as the Gd-IgA1deposited in IgAN. Complement activation in both the systemic circulation and the renal locality was much weaker in patients with MCD-IgAN than in those with non-MCD-IgAN. Our study suggests that IgAN with MCD might be MCD with coincidental IgA deposition. We hope that all these findings might aid a better understanding of and provide a theoretical basis for intervention in MCD-IgAN in the future.

## Data availability statement

The raw data supporting the conclusions of this article will be made available by the authors, without undue reservation.

## Ethics statement

The studies involving human participants were reviewed and approved by the ethics committees of Beijing Anzhen Hospital. The patients/participants provided their written informed consent to participate in this study.

## Author contributions

W-yG and HC made substantial contributions to the study concept and design. W-yG drafted the manuscript. HC critically revised the manuscript, supervised the entire study, and gave final approval to the article. W-yG and H-rD performed the immunofluorescence in renal biopsies. W-yG performed the ELISAs. The renal biopsies were reviewed and the staining intensity from anonymized sections were graded by W-yG and L-jS independently. W-yG and NY conducted statistical analyses. X-yX, G-qW, Z-rZ, W-rC, and YL collected the primary data and treated all the patients. All authors contributed to the article and approved the submitted version.

## References

[1] D'Amico G. The commonest glomerulonephritis in the world: IgA nephropathy. Q J Med (1987) 64(245):709–27. 3329736

[2] Berthoux FC Mohey H Afiani A. Natural history of primary IgA nephropathy. Semin Nephrol (2008) 28(1):4–9. doi: 10.1016/j.semnephrol.2007.10.001 18222341

[3] Rodrigues JC Haas M Reich HN. IgA nephropathy. Clin J Am Soc Nephrol (2017) 12(4):677–86. doi: 10.2215/CJN.07420716 PMC5383386 28159829

[4] Sinnassamy P O'Regan S. Mesangial IgA deposits with steroid responsive nephrotic syndrome: Probable minimal lesion nephrosis. Am J Kidney Dis (1985) 5(5):267–9. doi: 10.1016/S0272-6386(85)80120-7 4003395

[5] Kim JK Kim JH Lee SC Kang EW Chang TI Moon SJ. Clinical features and outcomes of IgA nephropathy with nephrotic syndrome. Clin J Am Soc Nephrol (2012) 7(3):427–36. doi: 10.2215/CJN.04820511 PMC3302681 22223610

[6] Li H Lu W Li H Liu X Zhang X Xie L. Immune characteristics of IgA nephropathy with minimal change disease. Front Pharmacol (2021) 12:793511. doi: 10.3389/fphar.2021.793511 34975488 PMC8716750

[7] Matsukura H Miya K Arai M Miyawaki T Inaba S. Minimal change variants with mesangial IgA deposits. Clin Nephrol (2007) 68(5):337–8. doi: 10.5414/CNP68337 18044269

[8] Westhoff TH Waldherr R Loddenkemper C Ries W Zidek W Giet M. Mesangial IgA deposition in minimal change nephrotic syndrome: Coincidence of different entities or variant of minimal change disease? ClinNephrol (2006) 65(3):203–7. doi: 10.5414/CNP65203 16550751

[9] Suzuki H Kiryluk K Novak J Moldoveanu Z Herr AB Renfrow MB. The pathophysiology of IgA nephropathy. J Am Soc Nephrol (2011) 22(10):1795–803. doi: 10.1681/ASN.2011050464 PMC3892742 21949093

[10] Moldoveanu Z Wyatt RJ Lee JY Tomana M Julian BA Mestecky J. Patients with IgA nephropathy have increased serum galactose-deficient IgA1 levels. Kidney Int (2007) 71(11):1148–54. doi: 10.1038/sj.ki.5002185 17342176

[11] Zhao N Hou P Lv J Moldoveanu Z Li Y Kiryluk K. The level of galactose-deficient IgA1 in the sera of patients with IgA nephropathy is associated with disease progression. Kidney Int (2012) 82(7):790–6. doi: 10.1038/ki.2012.197 PMC3443545 22673888

[12] Suzuki H Yasutake J Makita Y Tanbo Y Yamasaki K Sofue T. IgA nephropathy and IgA vasculitis with nephritis have a shared feature involving galactose-deficient IgA1-oriented pathogenesis. Kidney Int (2018) 93(3):700–5. doi: 10.1016/j.kint.2017.10.019 29329643

[13] Suzuki H Fan R Zhang Z Brown R Hall S Julian BA. Aberrantly glycosylated IgA1 in IgA nephropathy patients is recognized by IgG antibodies with restricted heterogeneity. J Clin Invest (2009) 119(6):1668–77. doi: 10.1172/JCI38468 PMC2689118 19478457

[14] Wyatt RJ Julian BA. IgA nephropathy. N Engl J Med (2013) 368(25):2402–14. doi: 10.1056/NEJMra1206793 23782179

[15] Maillard N Wyatt RJ Julian BA Kiryluk K Gharavi A Fremeaux-Bacchi V. Current understanding of the role of complement in IgA nephropathy. J Am Soc Nephrol (2015) 26(7):1503–12. doi: 10.1681/ASN.2014101000 PMC4483595 25694468

[16] Haas M. Histology and immunohistology of IgA nephropathy. J Nephrol (2005) 18(6):676–80. 16358224

[17] Levey AS Stevens LA Schmid CH Castro AF 3rd Feldman HI Kusek JW. A new equation to estimate glomerular filtration rate. Ann Intern Med (2009) 150(9):604–12. doi: 10.7326/0003-4819-150-9-200905050-00006 PMC2763564 19414839

[18] Working Group of the International Ig ANN the Renal Pathology S Roberts IS Cook HT Troyanov S Alpers CE. The Oxford classification of IgA nephropathy: Pathology definitions, correlations, and reproducibility. Kidney Int (2009) 76(5):546–56. doi: 10.1038/ki.2009.168 19571790

[19] Guo WY Sun LJ Dong HR Wang GQ Xu XY Zhao ZR. Glomerular complement factor h-related protein 5 is associated with histologic injury in immunoglobulin a nephropathy. Kidney Int Rep (2021) 6(2):404–13. doi: 10.1016/j.ekir.2020.11.019 PMC7879122 33615066

[20] Barratt J Feehally J. Treatment of IgA nephropathy. Kidney Int (2006) 69(11):1934–8. doi: 10.1038/sj.ki.5000419 16641928

[21] Li XW Liang SS Le WB Cheng SQ Zeng CH Wang JQ. Long-term outcome of IgA nephropathy with minimal change disease: A comparison between patients with and without minimal change disease. J Nephrol (2016) 29(4):567–73. doi: 10.1007/s40620-015-0242-9 26537358

[22] Cho WH Park SH Choi SK Jung SW Jeong KH Kim YG. Characterization of IgA deposition in the kidney of patients with IgA nephropathy and minimal change. J Clin Med (2020) 9(8):2619. doi: 10.3390/jcm9082619 32806730 PMC7464421

[23] Li XW Cheng SQ Liang SS Le WB Zeng CH Wang JQ. Comparison between patients with IgA nephropathy with minimal change disease and patients with minimal change disease. Clin Nephrol (2016) 85(5):273–81. doi: 10.5414/CN108727 26951969

[24] Wang J Juan C Huang Q Zeng C Liu Z. Corticosteroid therapy in IgA nephropathy with minimal change-like lesions: a single-centre cohort study. Nephrol Dial Transplant (2013) 28(9):2339–45. doi: 10.1093/ndt/gft211 23787555

[25] Qin J Yang Q Tang X Chen W Li Z Mao H. Clinicopathologic features and treatment response in nephrotic IgA nephropathy with minimal change disease. Clin Nephrol (2013) 79(1):37–44. doi: 10.5414/CN107682 22948116

[26] Shen H Gu W Mao J Zhu X Jin X Fu H. Clinical characteristics of concomitant nephrotic IgA nephropathy and minimal change disease in children. Nephron (2015) 130(1):21–8. doi: 10.1159/000382035 25924689

[27] Ji S Liu M Chen J Yin L Sha G Chen H. The fate of glomerular mesangial IgA deposition in the donated kidney after allograft transplantation. Clin Transplant (2004) 18:536–40. doi: 10.1111/j.1399-0012.2004.00206.x 15344956

[28] Suzuki K Honda K Tanabe K Toma H Nihei H Yamaguchi Y. Incidence of latent mesangial IgA deposition in renal allograft donors in Japan. Kidney Int (2003) 63:2286–94. doi: 10.1046/j.1523-1755.63.6s.2.x 12753320

[29] Gharavi AG Kiryluk K Choi M Li Y Hou P Xie J. Genome-wide association study identifies susceptibility loci for IgA nephropathy. Nat Genet (2011) 43(4):321–7. doi: 10.1038/ng.787 PMC3412515 21399633

[30] Guo WY Zhu L Meng SJ Shi SF Liu LJ Lv JC. Mannose-binding lectin levels could predict prognosis in IgA nephropathy. J Am Soc Nephrol (2017) 28(11):3175–81. doi: 10.1681/ASN.2017010076 PMC5661287 28698271

[31] Guo WY Liu QZ Zhu L Li ZY Meng SJ Shi SF. Coding and noncoding variants in CFH act synergistically for complement activation in immunoglobulin a nephropathy. Am J Med Sci (2018) 356(2):114–20. doi: 10.1016/j.amjms.2018.04.006 30219152

[32] Kim SJ Koo HM Lim BJ Oh HJ Yoo DE Shin DH. Decreased circulating C3 levels and mesangial C3 deposition predict renal outcome in patients with IgA nephropathy. PLoS One (2012) 7(7):e40495. doi: 10.1371/journal.pone.0040495 22792353 PMC3391269

